# Prevalence of Skeletal Fluorosis in Northern Tanzania: A Follow-Up Study

**DOI:** 10.9745/GHSP-D-22-00342

**Published:** 2023-12-22

**Authors:** Anna Foat, Claire Stevens, Grace George, John Massawe, Ally Mhina, William K. Gray, Blandina T. Mmbaga, Deogratias S. Rwakatema, Paul Sallis, Helen Jarvis, Irene Haule, Daniel Benedict, Richard Walker

**Affiliations:** a Faculty of Medical Sciences, Masters by Research, Newcastle University, Newcastle Upon Tyne, United Kingdom.; b Hai District Hospital, Boma Ng'ombe, United Republic of Tanzania.; c Northumbria Healthcare NHS Foundation Trust, North Tyneside General Hospital, North Shields, United Kingdom.; d Kilimanjaro Clinical Research Institute, Moshi, United Republic of Tanzania.; e School of Engineering, Newcastle University, Newcastle Upon Tyne, United Kingdom.; f Population Health Science Institute, Newcastle University, Newcastle Upon Tyne, United Kingdom.; g Hai District Water Authority, Boma Ng'ombe, United Republic of Tanzania.

## Abstract

**Objectives:**

Skeletal fluorosis is a metabolic bone disease caused by excessive exposure to fluoride, predominantly through contamination of drinking water. This study aimed to identify all cases of skeletal fluorosis in Tindigani village situated in Northern Tanzania. This was done following changes in drinking water sources after a previous prevalence study in 2009 in this population.

**Methods:**

In a door-to-door cross-sectional study of Tindigani village, a sample of residents was assessed for skeletal fluorosis and dental fluorosis. Diagnosis of skeletal fluorosis was based on pre-defined angles of deformity of the lower limbs. Dental fluorosis was diagnosed and graded using the Thylstrup and Fejerskov Index. Samples from current drinking water sources underwent fluoride analysis.

**Results:**

Tindigani village had a population of 1,944 individuals. Of the 1,532 individuals who were screened, 45 had skeletal fluorosis, giving a prevalence of 3.3% (95% confidence interval [CI]=2.4, 4.3). Dental fluorosis was present in 82.5% of those examined (95% CI=79.8, 85.3). Dental fluorosis was present in all individuals with skeletal fluorosis and at higher grades than in the rest of the population. Drinking water samples were collected from 28 sources. These included piped, surface, well, and borehole water sources. Fluoride concentrations ranged from 0.45–38.59 mg/L of fluoride.

**Conclusions:**

Skeletal fluorosis is an ongoing but preventable health problem in the current population. The delivery of sustainable low fluoride piped water to this community would be of clear health benefit. This has been addressed at a local level.

## Abstract (precis)

This follow-up study uniquely identifies the incidence rates for skeletal fluorosis in Tindigani village in Northern Tanzania and suggests that skeletal fluorosis in this population is an ongoing, yet preventable cause of long-term disability requiring public health intervention.

## Teaser

**Key Findings:**

- Nine years after an initial prevalence study in Tindigani village in Northern Tanzania and following increased use of low fluoride piped drinking water, skeletal fluorosis (SF) is still an ongoing issue, as evidenced by the emergence of new cases.
- Although the etiology of SF is complex and multifactorial, fluoride exposure through drinking water appears to be the largest contributing factor, with high levels of fluoride found in well and borehole water sources.
- Of the individuals screened, 3.3% had SF, a decrease from 4.4% in 2009.

**Key Implications:**

- Provision of low fluoride water sources requires collaboration between public health officials and local water authorities.
- There is a need to raise awareness about the causes and prevention of SF among the population in this area, as well as among public health officials, local water authorities, and health care professionals.
- This study can be used to highlight the importance of low fluoride piped water and may be a reproducible model for other endemic areas in Tanzania and other countries along the East African Rift Valley that have high fluoride water sources.

## INTRODUCTION

Fluorosis affects around 70 million people worldwide^1^ but remains a neglected public health concern.^2^ The East African Rift Valley system, which traverses Tanzania, is a well-documented high fluoride area^1^^,^^3^^,^^4^ with fluoride contamination of ground and surface drinking water sources from underlying rock.^1^^,^^5^^–^^9^ Drinking water is the largest single contributor to daily fluoride intake.^1^^,^^8^ The United Nations Sustainable Development Goal 6 aims to achieve universal access to safe drinking water by 2030 and references fluoride as a priority chemical for water quality testing.^10^ Fluoride is unevenly distributed in ground and surface water, both vertically and horizontally.^8^ This makes mapping low fluoride areas exceptionally difficult, and water quality testing for excessive fluoride concentration is often not undertaken.^5^

Once ingested, fluoride is incorporated into calcium-rich areas, including the dental and skeletal systems.^1^^,^^11^ Low concentrations of fluoride in drinking water, 0.5–1 mg/L, are associated with cariostatic benefits.^12^ This is particularly seen in children^1^^,^^12^^,^^13^ and has shown improved skeletal health.^3^ The World Health Organization (WHO) recommends that the permissible upper limit of drinking water fluoride is 1.5 mg/L (equivalent to 1.5 parts per million).^3^^,^^8^^,^^9^^,^^14^^,^^15^ However, it is predicted that 30%^3^^,^^16^ of drinking water sources in Tanzania are above the WHO recommended limit. Intake exceeding 1.5 mg/L is associated with negative health impacts.^3^^,^^6^^,^^17^ These fall under the umbrella term of endemic fluorosis,^11^^,^^15^ which encompasses dental fluorosis (DF) and skeletal fluorosis (SF). DF causes marked pitting^1^ and discoloration^8^ of the teeth. Although changes are largely cosmetic, the teeth become brittle and mastication difficulties may arise.^18^ The Thylstrup and Fejerskov Index (TFI) is used to accurately grade DF from normal enamel translucency with no evidence of fluorosis (grade 0) to loss of the main part of enamel with changed anatomical appearance (grade 9).^19^

SF (osteofluorosis^3^ or hydric fluorosis^11^) is a chronic metabolic bone disease^20^ due to excessive exposure to higher levels of fluoride, 4–8 mg/L.^1^^,^^3^^,^^8^^,^^21^ Intake over 10 mg/L is linked to crippling SF,^1^ associated with profound immobility.^3^^,^^16^ SF is characterized by a combination of osteosclerosis, osteoporosis, osteomalacia, exostosis,^7^^,^^11^^,^^18^^,^^22^ calcification of ligaments and tendons, and extreme bone deformity.^1^ The cervical and lumbar vertebrae and major joints of the extremities^11^ are most severely affected and can lead to pathological fractures.^23^ Characteristic deformities include genu varum, genu valgum, and saber tibia.^11^ Neurological complications occur secondary to skeletal changes as a result of mechanical compression of nerves.^23^ Juvenile SF, also known as Kenhardt bone disease,^11^^,^^24^^,^^25^ describes the condition in children and adolescents. Radiology offers the best method for confirmation of diagnosis in SF through identification of osteophytes at muscular insertion sites^11^ and exclusion of active rickets, which presents with a similar clinical picture.^25^ Serum fluoride measurements rarely help with diagnosis.^11^ Without radiology, there is no specific grading system for SF and diagnosis is more difficult.

There is no established treatment for fluorosis.^11^^,^^20^^,^^26^ Management focuses on symptom alleviation,^14^ orthopedic intervention,^27^ and prevention of further exposure. In remote areas, individuals are forced to live with severe deformities, often unable to walk without pain. Unfortunately, many initiatives for defluoridation have failed,^1^^,^^28^ and most are expensive and inefficient in rural communities.^3^^,^^29^ One that has been used locally in Tanzania is filtration of water through bone char, but this process affects the water's taste, which many people do not find palatable.^30^ The filters come in different sizes, such as for a family or whole village. However, when the fluoride level in the water is very high, the bone char may need to be replaced frequently, which can prove limiting.^30^ Exposure to sunlight, with resultant evaporation of water and collection of condensate, can also be used to lower fluoride levels, but this is very cumbersome in anything but small volumes.^31^ The most effective long-term solution to minimize fluoride toxicity is the provision of sustainable, low-fluoride piped water to affected areas. Although fluorotic enamel changes cannot be reversed,^18^ SF is regarded as partly reversible, as damaged osseous tissue is replaced when fluoride ingestion stops.^18^

There is no established treatment for fluorosis, and management focuses on symptom alleviation, orthopedic intervention, and prevention of further fluoride exposure.

It is not fully understood why individuals in the same environment with the same exposure to the same level of fluoride show different severities of or apparent total absence of SF.^15^ Links to low body mass index (BMI),^18^ the use of trona or magadi (a fluoride rich food tenderizer),^32^^,^^33^ and potential protective effects of estrogen^11^ have all been demonstrated. However, the full effects of these variables on the pathogenesis of the disease are still unknown. Recent research from Ethiopia supports the premise that low dietary calcium intake is significantly associated with SF among women^22^^,^^34^ and school-aged children.^28^

There are no published incidence rates for SF in sub-Saharan Africa. In 2004, SF was identified in Tindigani village, which is within a demographic surveillance site in the Hai district of Northern Tanzania.^4^^,^^24^ In 2009, a case-control study assessed the prevalence and etiology of SF within Tindigani village^24^ and identified 56 cases of SF. The population previously relied on river water, wells (generally dug by hand), and boreholes (mechanically dug) provided by nongovernmental organizations (NGOs).^4^^,^^24^

After the 2009 original prevalence study, residents were educated about the risks of high fluoride and informed of sources with the highest concentrations, namely borehole pumps and wells. The Hai District Water Authority installed piped water to Tindigani village in 2010, but there have been problems with maintaining the supply. Residents often face a long commute on foot to neighboring villages with piped water, as well as financial constraints (US$0.20 per 20 L).^4^

This follow-up study aimed to assess SF in Tindigani village 9 years after the original prevalence study was conducted to gain an accurate estimate of the prevalence of clinically visible SF. As a follow-up study, this is the first of its kind to accurately calculate the incidence rate of SF in a population in sub-Saharan Africa.

## METHODS

### Design

A cross-sectional, door-to-door prevalence study with selective sampling of drinking water sources.

Data collection occurred between March and May 2018. This was undertaken by AF and CS, assisted by 2 Tanzanian assistant medical officers (JM, AM). A village enumerator assisted with translation between Swahili and local tribal languages. This study used recent census data (2018) for background demographic details.

### Setting

This study was conducted in Tindigani village in the south of the Hai district of Northern Tanzania.

### Door-to-Door

The lead member of each household confirmed the household name along with the name, age, and sex of all occupants. Signs of clinically visible SF were described to identify possible cases of SF in absent household members. Where SF was presumed or queried in an absentee, the household was revisited to allow for musculoskeletal assessment. This sampling method allowed all members of the population to be screened for SF, aiming for the majority to be assessed in person.

### Village School and Kindergarten

Visits to the village school and kindergarten identified children absent at the time of door-to-door assessments. Name, age, and household name were taken from registers to allow matching of children to households.

### SF

Data collectors were trained by HJ to perform a musculoskeletal assessment to identify and quantify clinically visible SF, reflecting methods used in the original prevalence study. A 12-inch protractor goniometer was used to measure coronal tibiofemoral (CTF) angles. Genu varum (bow legs) is defined as a CTF angle of more than 7 degrees, and genu valgum (knock knees) is present when the angle is negative.^24^ Participants were also screened for saber tibia (anterior convexity of the tibia). Identifiers (name, age, and household name) of those with SF were matched to a case list to identify follow-up cases from the 56 identified in the original prevalence study.^24^

### DF

A dental specialist, PM, provided training in assessment and dental photography. Dental examination of the mandibular central incisors identified DF. Consenting participants had photographs taken using a Nikon D90 camera. Photographs were graded using TFI, with a proportion verified by a dental specialist (PM).

### Water Sources

Information on current and historical drinking water sources was obtained. Noting historical drinking water sources, specifically those in 2009, allowed for comparison over time.

A sample from accessible drinking water sources was taken for analysis. This systematically selective nature of sampling has been used in previous studies.^1^ Samples were collected in 30 ml polypropylene containers. It was labeled at the sample site and transferred to the United Kingdom for analysis at Newcastle University School of Engineering. Sample analysis involved a Thermo Scientific Dionex Integrion HPIC unit with 5 concentrations of fluoride for the standard calibration curve (0.5, 2, 5, 10, and 20 parts per million), as used in previous studies.^35^

### Statistical Analysis

Data underwent statistical analysis using SPSS Version 24 for Windows. Confidence intervals for prevalence and proportions were calculated based on the assumptions of the binomial distribution. In bivariate analysis, the Mann-Whitney U test and chi-square test were used to assess significance for continuous and categorical variables, respectively. A binary regression model, with backward stepwise (likelihood ratio), was used to adjust the relationship between SF and other potential confounders in the study. The dependent variable was the presence of SF. The independent variables were age, BMI, and TFI grade. Statistical significance was fixed at a *P* value of less than .05.

### Ethical Approval

Ethical approval was granted by the National Institute for Medical Research, Tanzania, and the Kilimanjaro Christian Medical University College. Participation in the study was voluntary and without compensation.

## RESULTS

**FIGURE 1:**
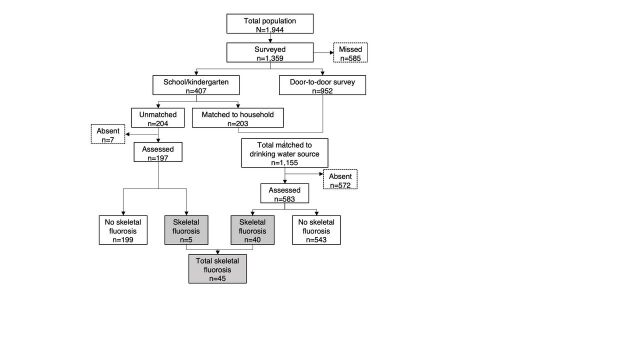
Flow Chart to Show Identified Cases of Skeletal Fluorosis, Tindigani, Tanzania

Of the total 1,944 Tindigani population, 1,359 (69.9%) were screened via door-to-door and school and kindergarten visits (Figure 1). Children seen at school and kindergarten were matched to households in 49.9% of cases. Of the 1,359 screened, 671 (49.4%) were male, giving a male-to-female ratio of 1:1.03, similar to the male-to-female ratio of 1:0.97 in the total population.

### Prevalence of SF

Of the 1,359 screened, 780 (57.4%) were assessed, and 45 cases of SF were identified (Figure 1). Therefore, the prevalence of SF in all individuals screened is 3.3% (95% confidence interval [CI]=2.4, 4.3), compared to 4.4% (95% CI=3.3, 5.6) in 2009.^24^

Age-specific prevalence rates show that SF was most common in those aged 40–44 years (Table 1). The highest number of SF cases (n=12) was seen in those aged 10–14 years. SF cases in the original prevalence study ranged from 2 to 30 years. When using that same age range in this study, the 15–19 years group had the highest prevalence of SF, compared to 10–14 years in the original prevalence study.^24^

**TABLE 1.** Age-Specific Prevalence Rates of Skeletal Fluorosis, Tindigani, Tanzania

Age Range, Years	Cases, No.	Study Population, No.	Prevalence, % (95% CI)
0–4	1	260	0.38 (0, 1.1)
5–9	7	344	2.0 (0.5, 3.5)
10–14	12	229	5.2 (2.4, 8.1)
15–19	6	84	7.1 (1.6, 12.7)
20–24	5	91	5.5 (0.8, 10.1)
25–29	5	80	6.3 (0.9, 11.6)
30–43	2	66	3.0 (0, 7.2)
35–39	0	36	0
40–44	3	31	9.7 (0, 20.1)
45–49	2	34	5.9 (0, 13.8)
50–99^a^	2	104	1.9 (0, 4.6)
Total	45	1359	3.3 (2.4, 4.3)

Abbreviation: CI, confidence interval.

a Age bands combined due to low numbers of cases in each age range. 95% confidence interval (CI) negative values rounded to 0.

### Incidence of SF

Of the 45 cases identified, 18 were new cases and the remaining 27 were follow-up cases identified from the original prevalence study.^24^ Therefore, the incidence rate for SF in this population since 2009 was 1.69 per year per 1,000 population.

Of the 45 cases of SF identified, 18 were new cases and the remaining 27 were follow-up cases identified from the original prevalence study.

### DF

**FIGURE 2:**
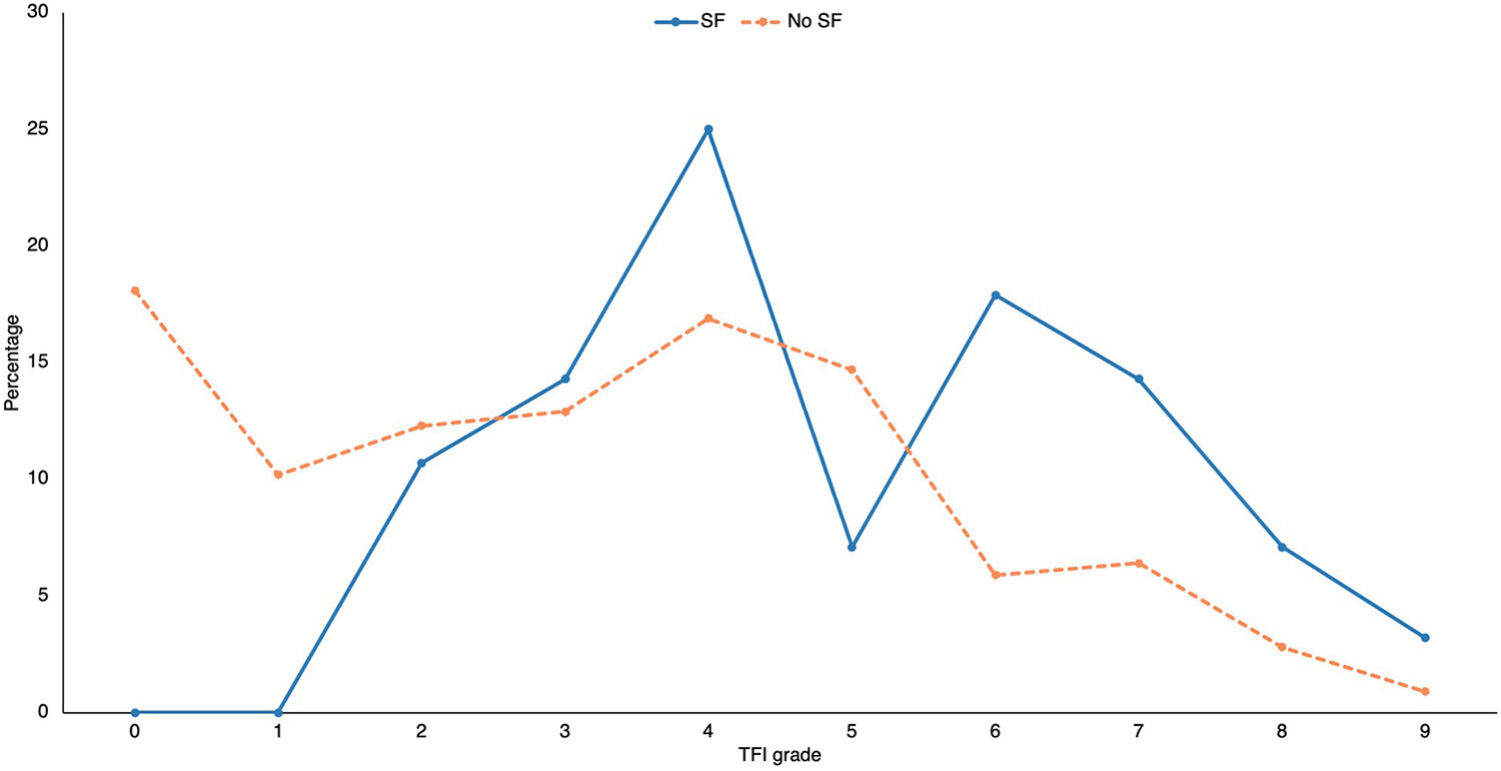
Percentage per TFI Grade Split by Presence of Skeletal Fluorosis, Tindigani, Tanzania Abbreviations: SF, skeletal fluorosis; TFI, Thylstrup and Fejerskov Index.

TFI grades were ascertained for 750 (55.2%) of those screened. DF was endemic in the village, seen in 619 (82.5%; 95% CI=79.8, 85.3) individuals. DF was present in all cases with SF and of a higher grade in comparison to the rest of the population (Figure 2). There were no instances where an individual with SF had a TFI grade below grade 2, with the majority having a TFI grade 4 and above. A higher percentage of those with SF, when compared to absence of SF, were seen with TFI grade 6 and above.

### Age

In multivariable binary logistic regression modeling, a significant association was seen between SF and age when TFI grade and BMI were controlled (1.403; 95% CI=1.222, 1.609; *P*<.001).

### Comparison of Those With and Without SF

This study found no significant difference between sexes (*P*=.805) regarding the presence of SF but did show a significant difference in mean age (*P*=.001) and mean TFI grade (*P*<.001) (Table 2).

**TABLE 2.** Comparison Statistics Between Skeletal Fluorosis and No Skeletal Fluorosis, Tindigani, Tanzania

	Skeletal Fluorosis	No Skeletal Fluorosis	Statistical Test Used	Significance
Mean age	21.93	17.36	Mann-Whitney U	*P*=.001(U=2,109.5)
Male-to-female ratio	23:22	647:667	Pearson Chi-Square	*P*=.805(χ^2^=0.651)
Mean Thylstrup and Fejerskov Index grade	4.96	3.21	Mann-Whitney U	*P*<.001(U=5,935.5)

### Case Comparison With Original Prevalence Study

Of the 18 new cases, 15 were aged 2–30 years, and this range was used to allow direct comparison of data with the cases in the original prevalence study.^24^ Evidence of CTF angle abnormalities was found in 5 individuals, of whom 4 showed angles consistent with major genu varum and 1 showed major genu valgum. The remaining 10 cases (66.7%) had signs of genu varum but did not meet the greater than 7 degrees criteria.^24^

Compared to the 2009 original prevalence study, the effects of SF are less severe, shown by reduced mean CTF angles, no presence of saber tibia, and reduced impact on education (Table 3). However, SF is still an issue in this community, as evidenced by 20% of the new cases who felt unable to achieve daily tasks.

**TABLE 3.** Comparison Between 2009 Original Prevalence Skeletal Fluorosis Study and 2018 Case Findings, Tindigani, Tanzania

Finding	2009 (n=56)	2018 (n=15)
Mean Thylstrup and Fejerskov Index grade^a^	6	5
Mean coronal tibiofemoral angle in worse leg with genu varus	10.17	6.16
Mean coronal tibiofemoral angle in worse leg with genu valgus	−9.47	−3
Saber tibia, no. (%)	18 (32)	0 (0)
Education affected, no. (%)	17 (30)	1 (6.6)
Unable to achieve daily tasks, no. (%)	17 (30)	3 (20)

a Thylstrup and Fejerskov Index grade treated as a continuous variable.

### Drinking Water Sources

Of the total cohort, 1,155 (85%) participants were linked to 229 households with information about current and historical drinking water sources. Of these, 956 (82.8%) reported current use of piped drinking water from neighboring villages. The remaining 199 (17.2%) still used well (8.1%), surface (river or spring [6.0%]), and borehole (3.1%) water sources.

Furthermore, 1,080 of the 1,155 (93.5%) recalled their main source of drinking water in 2009, with 578 (53.7%) using a borehole installed by an NGO in 1999. During this study, this borehole pump was broken, so a sample could not be obtained. In 2009, only 5.8% used piped water, and those not using the NGO borehole used surface (30.1%) and well water (10.4%).

**FIGURE 3:**
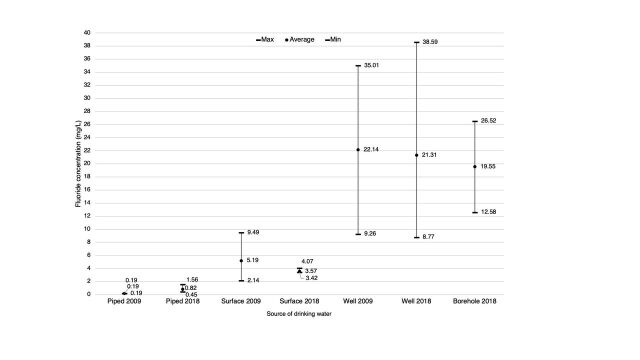
Change in Fluoride Concentration of Different Drinking Water Sources Between 2009 and 2018, Tindigani, Tanzania

A total of 28 samples were collected from accessible current drinking water sources within Tindigani village and the neighboring villages, Sanya Station and Rundugai. Fluoride concentrations from all samples were compared to levels seen in the 2009 study to assess change over time (Figure 3). The average concentration of fluoride in samples from surface and well water has decreased since 2009.^24^ Interestingly, the concentrations taken from piped sources have slightly increased, with 1 sample exceeding the WHO recommendation by 0.06 mg/L. However, all piped water samples remain well below the level that poses a risk of SF.

At school, children were accessing an NGO borehole installed in 2016 that had a fluoride concentration of 12.6 mg/L, over 8 times the recommended value from WHO.^9^^,^^13^

## DISCUSSION

This follow-up study reassessed a population 9 years after an initial prevalence study following changes to drinking water sources. As a follow-up study, this is the first of its kind to calculate the incidence rate of SF in a population in sub-Saharan Africa and found the incidence to be 1.69 per year per 1,000 population.

This study is the first of its kind to calculate the incidence rate of SF in a population in sub-Saharan Africa.

The use of low fluoride piped water has increased from 5.8% in 2009 to 82.8% in 2018, yet access is restricted due to financial constraints and a lengthy commute to neighboring villages on foot, averaging 4 hours for a return trip. Accessibility limits not only the elderly and the sick but will mean all members of the population might use unsafe, high fluoride drinking water due to ease of access.

This study has identified high levels of fluoride in well and borehole water sources. Unlike wells, NGO-installed boreholes provide protection against pathogenic contamination, though this study highlights that boreholes pose an increased risk in terms of fluoride concentration. Although the majority of households now collect low fluoride water from piped sources, schoolchildren are still exposed to high fluoride water from an NGO borehole during the school day.

The current prevalence of SF in this population is 3.3% (95% CI=2.4, 4.3) and has decreased from 4.4% in 2009 (95% CI=3.3, 5.6),^24^ likely due to the increased use of piped drinking water. These prevalence rates are much lower when compared to other countries: 22.3%^21^ in India and 26.2% in a study of a similar size in China.^20^ However, all those affected by SF are left with lifelong disabling deformities and may be unable to work or contribute to wider society. In this community, work involves manual labor in farming, herding cattle, and fetching firewood and water. When comparing new cases of SF to cases identified in the original prevalence study, the effects of SF were seen to be less severe, shown by reduced mean CTF angles and absence of saber tibia. However, SF still remains an ongoing issue impacting an individual's ability to perform daily tasks.

SF cases in the original prevalence study ranged from 2 to 30 years. When using this same age range in this study, 15–19 years has the highest prevalence of SF compared to 10–14 years in the original prevalence study.^24^ One of the boreholes with highest levels of fluoride was set up in 1999, and therefore people aged older than 19 years would not have been affected by this water source during childhood. This would have affected younger people in the original prevalence study.

A significant association was seen between SF and age when TFI grade and BMI were controlled for (1.403; 95% CI=1.222, 1.609; *P*<.001). This finding makes logical sense, as with increasing age, the individual has been exposed to high fluoride consumed in drinking water for a longer period of time and is in keeping with other studies.^20^^,^^22^^,^^28^

Previous studies identify estrogen as a protective factor for SF,^11^ with lower rates of SF seen in females.^11^^,^^24^ This study found no significant difference between sexes. The reason for this is unknown, although exposure to higher concentrations of fluoride in this environment may surpass the protective effects of the hormone and may explain why females are also affected in this population.

It has been shown that increased dietary calcium may mitigate the severity of SF, and it is important to consider promoting high-calcium foods or supplements that are cost-effective and sustainable, such as calcium-containing eggshell powder supplementation, which has been tested in Ethiopia.^22^^,^^28^^,^^34^

A major advantage of this study is the large sample size. The reliability of the demographic data is strengthened by the comparison of results from the recent census within this established demographic surveillance site. The use of door-to-door assessment with school and kindergarten assessments provided 2 overlapping data sources, and data entry was reviewed for duplicates and omissions.

This study produced unique follow-up data for cases with SF. Longitudinal interpretation of changes in fluoride concentration over time were based on robust water fluoride level analysis. The use of analogous methods allows direct comparison of results to those obtained from the original prevalence study.^24^

### Limitations

A major limitation of this study is that 30.1% of the population was not seen due to inaccessibility of certain areas during the wet season. However, as passable areas changed over time, it is unlikely a whole area of individuals with different findings would have been identified. As a result, the prevalence rate of 3.3% is likely representative of the whole population. Studies have shown seasonal variations in fluoride levels,^32^^,^^36^ with higher levels reported in dry seasons,^3^^,^^32^ due to dilution effects exerted by rainfall.^3^ Therefore, the concentrations obtained in this study may be an underestimation.

Although Tindigani is a demarcated village within a demographic surveillance site, the village boundaries may have changed as the population increased in size. Every effort was made to ensure those in the study had lived in Tindigani village for their whole lives to obtain an accurate comparison to the 2009 study.

Door-to-door assessments relied on members of households to identify clinically visible SF in an absentee, which was deemed appropriate as there was existing knowledge about SF in the population following the original prevalence study. When SF was presumed or queried in an absentee, the household was revisited. This was particularly important as nomadic cattle herding was common in this area, resulting in multiple members of the household, particularly males, being absent at any given time.

Unlike the TFI for DF, SF lacks a robust grading system without the use of radiology, which has also been a limitation in other studies.^34^ Consequently, diagnosis in the field relies on the use of CTF angles for grading the severity of genu varum and genu valgum deformities. CTF angles change with development of the bones in the lower limbs.^37^^,^^38^ Genu varum is maximal at 6 months^39^ yet abnormal beyond the age of 2 years.^37^^,^^39^^,^^40^ Physiological genu valgum, maximal at 4 years,^39^ can persist up to the age of 7 years.^37^ Both genu varum and valgum should correct spontaneously^38^ to a mean of “less than 6 degrees” and to a “neutral (0 degree)” CTF angle,^39^ respectively. CTF angles have been quoted as the accurate way to quantify angulation.^40^ However, the use of CTF angles alone cannot rule out active rickets as a differential diagnosis,^25^ and CTF angles may only identify gross clinically visible limb abnormalities, unlike radiographs, which can identify early SF. Therefore, it is likely SF is under-reported in this study. As all individuals with SF had DF and their DF was of a higher grade in comparison to the rest of the population, DF may highlight an area suitable for diagnostic screening for those at risk of SF.

Another limitation was the use of recall. Reports of historical water sources used by households are potentially subject to recall bias. It cannot be confirmed that the high fluoride drinking water is the only factor causing SF in this population. Other factors may interact and contribute to SF, and studies have shown the contribution from food can exceed that from water.^13^ Ethiopian studies have shown calcium deficiency exacerbates symptoms of SF,^22^^,^^28^ and supplementation in the form of calcium-containing eggshell powder has been shown to be beneficial.^34^ Other influencing factors have been described, including social demographics, activity levels, health status,^15^ and genetics.^4^^,^^20^ These additional factors may go some way towards explaining why some are severely affected with crippling deformities while others are seemingly unaffected.

## CONCLUSIONS

This follow-up study uniquely identifies the incidence rates for SF in this area and suggests that SF in this population is an ongoing issue. It is the first study in sub-Saharan Africa to measure the incidence of SF. Although the etiology of SF is complex and multifactorial, fluoride exposure through drinking water appears to be the largest contributing factor. The use of low fluoride piped water in Tindigani has increased significantly, yet despite this, SF is still an issue within this population, as evidenced by the emergence of new cases. SF can lead to lifelong disability from an early age. Affected children miss time at school due to pain preventing them from walking. Without higher education, these children are limited to manual labor in subsistence farming or cattle herding, both of which may be intolerable due to the joint deformities caused by SF.

NGO-installed boreholes used historically and at present were shown to have fluoride concentrations well above the WHO-recommended standard. This calls for increased awareness regarding fluoride testing when establishing the water source and regularly thereafter, as fluoride levels may vary over time. Residents should be advised to reduce their exposure to high concentrations of fluoride by accessing piped water. However, in this low-resource setting, it is difficult to prohibit the use of well or borehole sources, as these save individuals time, energy, and money. Some members of the population have no choice but to use high fluoride sources for drinking water due to financial constraints.

Although local solutions may be feasible, it is important to look at the wider problem, as SF affects many communities in East Africa in the Rift Valley region, including in Ethiopia, Kenya, and Tanzania. Large-scale solutions such as piping water with safe fluoride levels from other areas could have a major impact, but in exceptionally rural areas, this is very challenging. Other measures, such as promoting high-calcium foods or low-cost dietary calcium supplementation (e.g., eggshell powder), have been shown to help mitigate symptoms of SF.

The response to the ongoing issue with SF should not be exclusively based on future research. It can be predicated that SF will continue to be an issue within Tindigani without affordable, low fluoride piped water within a reasonable commute of households. This has been addressed by the government at a local level following maintenance and reinstallation of accessible, low fluoride piped water from Uroki Bomang'ombe Water Supply and Losaa KIA Water Supply. Ensuring continued access to sustainable, low fluoride piped water in the long term is paramount to prevent potentially crippling SF in the future in this community.

There is a need to raise awareness about the causes and prevention of SF among the population in these areas, as well as among public health officials, local water authorities, and health care professionals.

This study can be used to highlight the importance of low fluoride piped water and may be a reproducible model for other endemic areas in Tanzania and other countries along the Rift Valley with high fluoride water sources.

## Notes

**Peer Reviewed**

**First published online:** XXX.
